# Integrating Additive and Traditional Manufacturing for Multiscale Bone Tissue Engineering Scaffolds

**DOI:** 10.3390/jfb16090349

**Published:** 2025-09-16

**Authors:** Yixuan Zhu, Haotian Gao, Qingchen Qiao, Yafei Yuan, Dongyu Fang, Yuxing Bai, Qingsong Jiang

**Affiliations:** 1 School of Stomatology, Capital Medical University, Beijing 100070, China; kevinzhu6678@gmail.com (Y.Z.); gaoht2000@mail.ccmu.edu.cn (H.G.); qchjoe@163.com (Q.Q.); lit2fei@163.com (Y.Y.); fangdy@mail.ccmu.edu.cn (D.F.); 2 Department of Prosthodontics, Beijing Stomatological Hospital, Capital Medical University, Beijing 100070, China; 3 Department of Orthodontics, Beijing Stomatological Hospital, Capital Medical University, Beijing 100070, China

**Keywords:** bone tissue engineering, multiscale scaffolds, additive manufacturing, traditional manufacturing, hierarchical structure

## Abstract

Additive manufacturing (AM) has emerged as a cutting-edge technology for fabricating biomimetic scaffolds with controllable architectures and compositional diversity, showing great promise in the fields of bone tissue engineering (BTE) and regenerative medicine. However, due to limitations in printing resolution and single-process capabilities, AM alone struggles to replicate the complex multiscale hierarchical structures inherent in native bone. Traditional fabrication techniques provide valuable complementary strategies to address these limitations. This review systematically summarizes recent advances in the construction of heterogeneous scaffolds from a multiscale design perspective, encompassing macro-, meso-, and microscale approaches. Emphasis is placed on the integration of major AM techniques—such as extrusion-based and light-based printing—with conventional methods including freeze-drying, gas foaming, and electrospinning. Particular attention is given to emerging in situ fabrication strategies, such as in situ foaming and mineralization, which enable spatially resolved and functionally graded architectures. Furthermore, this review explores pathways for constructing multiscale-integrated scaffolds and examines the current challenges and opportunities in clinical translation. Collectively, this work provides a comprehensive framework to guide the development of next-generation bone tissue scaffolds with enhanced biological performance and translational potential.

## 1. Introduction

Human bone is a highly heterogeneous, multiscale structural system characterized by well-defined hierarchical and gradient organization across macroscopic, mesoscopic, and microscopic levels [1]. Due to the structural and functional complexity of bone, the effective repair of critical-sized bone defects caused by tumors, trauma, or infection has long posed a major clinical challenge in orthopedics and oral-maxillofacial surgery. Although autografts and allografts demonstrate superior regenerative outcomes compared to xenografts, their widespread application remains limited by donor scarcity, immune rejection, and postoperative complications [2]. Notably, a recent large-scale survey by Rupp et al. [3] covering over one million orthopedic procedures in Germany demonstrated a decade-long decline in autograft use, contrasted by a substantial rise in the application of allografts and biomaterial-based substitutes. This shift underscores the reduced reliance on autologous bone grafting in clinical practice. The rising demand for bone graft substitutes has driven innovation in tissue engineering, spurring the development of biomimetic scaffolds with precisely controlled composition and architecture that recapitulate the hierarchical organization of native bone, thereby enabling more effective bone regeneration [4,5].

Additive manufacturing (AM) technologies, owing to their notable advantages in personalized structural design and material adaptability, are emerging as a pivotal approach for fabricating next-generation biomimetic scaffolds for bone tissue engineering (BTE). However, limitations in current printing resolution and microstructural processing capabilities still pose significant technical barriers to the accurate reconstruction of complex architectures at meso- to microscale levels [6]. In contrast, conventional scaffold fabrication techniques for BTE—such as freeze-drying, gas foaming, and electrospinning—offer certain advantages in replicating micro/nano-scale architectures. However, they generally lack effective control over macroscopic structure, geometric boundaries, and configurational stability, making it difficult to meet the demands of personalized adaptation and structural reconstruction in complex bone defect sites [6]. Accordingly, current research is increasingly focused on multiscale synergistic strategies that integrate AM with conventional techniques, aiming to achieve continuous modeling and hierarchical control from the macroscopic geometry down to micro- and nanoscale features. This approach offers a more faithful reconstruction of the native heterogeneity in bone structure and function.

This review adopts a biomimetic perspective to systematically examine the structural characteristics of native bone across the macro-, meso-, and microscale levels. It provides a comprehensive overview of AM techniques, processing methods, and scaffold construction strategies suited to each scale. Special attention is given to the recent advances in emerging 3D printing approaches and their integration with conventional fabrication techniques for BTE applications. Finally, this review envisions the development of cross-scale manufacturing integration and the challenges of clinical translation, aiming to provide theoretical guidance and technical references for the structural design and clinical application of next-generation multiscale heterogeneous bone scaffolds.

## 2. Literature Search and Bibliometric Mapping

To systematically analyze the construction strategies of heterogeneous scaffolds for BTE, a comprehensive literature search was conducted using the Web of Science Core Collection (WoSCC) database. The search spanned publications from 2009 to 2025, employing keywords such as “scaffold,” “bone tissue,” “additive manufacturing,” “phase separation,” “gas foaming,” and “salt leaching,” among others. Only English-language peer-reviewed articles and reviews were included.

**Figure 1:**
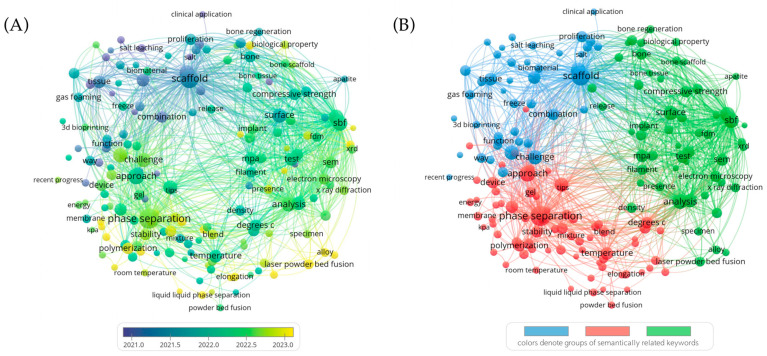
Keyword co-occurrence networks related to heterogeneous scaffold fabrication strategies for BTE. (**A**) Overlay visualization indicating the temporal evolution of research topics (color-coded by average publication year). (**B**) Clustered network visualization revealing three main thematic areas: biomaterial scaffold design (blue), microstructural regulation strategies (red), and functional evaluation (green).

Keyword co-occurrence networks were generated using VOSviewer (version 1.6.20) to visualize thematic structures and research hotspots. The resulting bibliometric maps (Figure 1) depict both the temporal evolution and thematic clustering of research topics. In the overlay visualization (Figure 1A), color gradients reflect the average publication year of each keyword: yellow nodes highlight emerging trends around 2023 (e.g., “phase separation,” “polymerization,” “Pickering emulsion”), whereas blue and green nodes correspond to earlier and ongoing themes such as “scaffold,” “bone regeneration,” and “surface.” In the clustered visualization (Figure 1B), keywords are grouped into three primary thematic clusters: biomaterial-based scaffold design (blue), microstructural regulation strategies (red), and performance evaluation or functional analysis (green). This clustering provided the conceptual framework for structuring subsequent sections of the review across macro-, meso-, and microscale levels.

## 3. Multiscale Structural Dimensions and Their Relevance to Bone Tissue Biomimicry

### 3.1. Structural Hierarchies of Materials

The hierarchical and multiscale classification of material structures exhibits diversity and complexity across different disciplines. Structural system in physics typically spans multiple levels, ranging from macroscopic to mesoscopic, microscopic, nanoscopic, femtoscopic, and even sub-femtoscopic scales. This scale hierarchy reflects the multilayered evolutionary mechanisms of material structures and functional properties, serving as a key to uncovering the fundamental nature of material behavior [7,8,9]. Among these, the “mesoscopic” scale refers to structures that bridge the gap between the microscopic and macroscopic levels, typically spanning from 10^−9^ to 10^−7^ meters. Owing to its significant overlap with the structural dimensions studied in nanoscience, this regime is often collectively referred to as “mesoscopic physics and nanoscience” [10].

In the context of biomimetic medicine and biomedical engineering, the classification of structural scales tends to focus more on functional biological response mechanisms. Studies have shown that materials with different structural scales can significantly induce distinct biological effects [11]. Understanding and appropriately aligning these distinct structural scales with their corresponding biological functions forms the foundation for achieving precise biomimetic design [12]. Specifically, the macroscopic scale refers to structures visible to the naked eye, typically larger than 100 μm, and primarily governs structural support and mechanical properties. The mesoscale encompasses microstructures ranging from 1 to 100 μm and submicron structures between 100 nm and 1 μm, which play a decisive role in cell adhesion and migration. In contrast, the nanoscale (<100 nm) is believed to regulate cell differentiation, gene expression, and tissue regeneration, as its dimensional range closely matches that of extracellular matrix (ECM) components such as collagen, proteins, and cell membrane receptors [13].

### 3.2. Hierarchical Scales of Bone Structure and Their Physiological Significance

**Figure 2:**
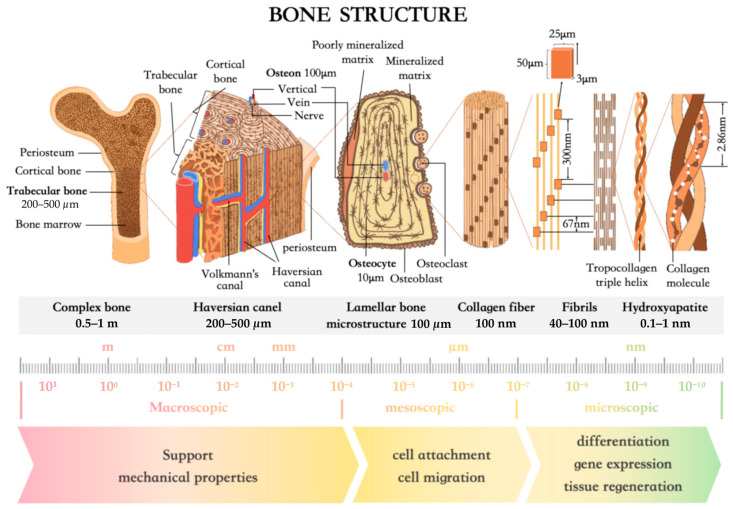
Schematic illustration of bone structures at different hierarchical levels and the biological implications of various scales in tissue engineering.

In the skeletal system, bone tissue is composed of three primary components at the macroscopic level, as is depicted in Figure 2: bone matrix, periosteum, and bone marrow. The bone matrix can be further categorized based on its density into cortical bone and trabecular bone. Trabecular bone exhibits a highly complex internal architecture formed by interwoven trabeculae, which are typically plate-like or rod-like in shape, with diameters ranging from approximately 100–500 μm and lengths reaching 1–2 mm depending on anatomical location and mechanical loading conditions, forming the structural basis together with cortical bone for the overall stiffness and strength of bone tissue [14].

At the mesoscale, the bone’s hierarchical structure is exemplified by the osteon, also known as the Haversian system, which constitutes the fundamental structural and functional unit of cortical bone. Osteons are cylindrical in shape and aligned longitudinally along the axis of long bones, situated between the inner and outer lamellae. Each osteon consists of 5 to 20 concentric lamellae encircling a central Haversian canal [15]. Transversely oriented Volkmann’s canals interconnect multiple Haversian canals, forming a three-dimensional vascular network that sustains bone metabolism and function [16]. The architectural organization of osteons also determines the spatial distribution of osteocytes, which extend dendritic processes through canaliculi, establishing an intricate intercellular communication network [17,18]. This mesoscale architecture plays a critical role not only in maintaining osteocyte viability but also in supporting the microenvironment for bone formation, remodeling, and mechanical responsiveness.

At the microscale, the bone matrix is composed of both organic and inorganic components, conferring superior mechanical performance and bioactivity. Approximately 90% of the organic matrix consists of type I collagen fibers, with the remainder comprising non-collagenous proteins such as bone morphogenetic proteins (BMPs) and proteoglycans, which contribute to matrix mineralization and remodeling [19,20]. Collagen molecules self-assemble into triple-helical structures, which further bundle into fibrils with diameters around 100 nm. The inorganic phase is primarily composed of hydroxyapatite (HA) nanocrystals, which account for approximately 65% of the dry weight of bone and are oriented longitudinally along the collagen fibrils [21]. These nanoscale arrangements not only provide the compressive strength essential for bone function but also contribute to osteoconductivity and osseointegration during bone regeneration.

## 4. Inspiration from the Hierarchical Structure of Natural Bone for BTE Scaffold Design

At multiple hierarchical scales, the design of BTE scaffolds must integrate physiological functions and regenerative requirements across the macro-, meso-, and microscale levels.

At the macroscale, extensive efforts have been devoted to replicating the structural complexity of cortical bone. Advanced scaffold architectures—such as unit cell lattices and triply periodic minimal surface (TPMS) structures—have demonstrated that pore sizes within the range of 250–500 µm promote osteogenesis and vascularization [22]. However, excessively large pores or suboptimal structural configurations can substantially compromise mechanical strength [23,24]. Meanwhile, clinically used allografts and biomaterial-based scaffolds also reflect inspiration from the distinct structural hierarchies of cortical and cancellous bone. Cortical allografts, known for their high mechanical strength and load-bearing capability, are widely utilized in reconstructing long bone diaphyseal defects, spinal fusions, and peri-prosthetic repairs. In salvage revision arthroplasty, cortical allografts have been shown to provide reliable structural support, facilitate bone defect reconstruction, and achieve union rates exceeding 90% [25]. However, cortical allografts undergo relatively slow remodeling, relying primarily on creeping substitution—a process whereby osteoclast—mediated resorption precedes osteoblastic new bone formation—coupled with intramembranous ossification to achieve integration with host bone [26]. In contrast, cancellous allografts—with their high porosity and abundant osteoinductive matrix—are widely used for tasks such as defect filling, joint fusions, and maxillofacial augmentation [27]. Their interconnected trabecular structure promotes rapid cell infiltration, angiogenesis, and new bone formation. In parallel, guided bone regeneration (GBR) technology, which mimics the barrier function of the periosteum, creates a confined space at the scaffold surface to prevent soft tissue ingrowth. While GBR has been widely applied in maxillofacial defect repair, its translation to large segmental long bone defects remains in early clinical investigation [28].

At the mesoscale, the successful reconstruction of trabecular bone architecture requires coordinated design of pore structure, spatial cell distribution, vascularization, and neural integration. Microporosity (<10 μm), resembling the surface roughness of internal bone structures, has been demonstrated to enhance osteointegration [29]. Co-culture systems involving osteogenic cells (e.g., bone marrow mesenchymal stem cells, osteoprogenitors) and endothelial cells have demonstrated enhanced paracrine signaling that supports vascular formation, immune modulation, and microenvironmental homeostasis [30]. Notably, insufficient vascular and neural integration is often associated with reduced mechanical function and regenerative outcomes [31]. As a result, AM strategies are increasingly focusing on scaffolds with vascular and neurogenic guidance. Studies have shown that hollow-channel designs combined with angiogenic factors, such as vascular endothelial growth factor (VEGF), improve vascularization [32], while incorporation of neurotrophic factors, such as nerve growth factor (NGF) and brain-derived neurotrophic factor (BDNF), or conductive components facilitates coordinated neurogenically guided bone regeneration [33].

At the microscale, scaffolds that mimic the mineralized collagen architecture and the micro-/nano-topographies of the native ECM, such as pillars, pits and grooves, have been shown to significantly enhance cell adhesion, proliferation, and osteogenic differentiation [34,35]. Surface chemistry and elemental composition—such as incorporation of Li, Mg, or F, or surface functionalization with drugs exhibiting anti-inflammatory, antioxidant, or anti-osteoporotic effects—have been widely explored for functional enhancement [36]. With advances in low-temperature bioprinting techniques, bioactive molecules such as BMP-2 and VEGF can now be effectively incorporated into hydrogel-based scaffolds, enabling controlled release and spatiotemporal regulation of bone regeneration [37]. The integration of these structures and functions is crucial for efficient oxygen and nutrient transport, waste removal, maintenance of tissue homeostasis, and transmission of biochemical, mechanical, and electrophysiological signals within the scaffold micro environment [38].

**Figure 3:**
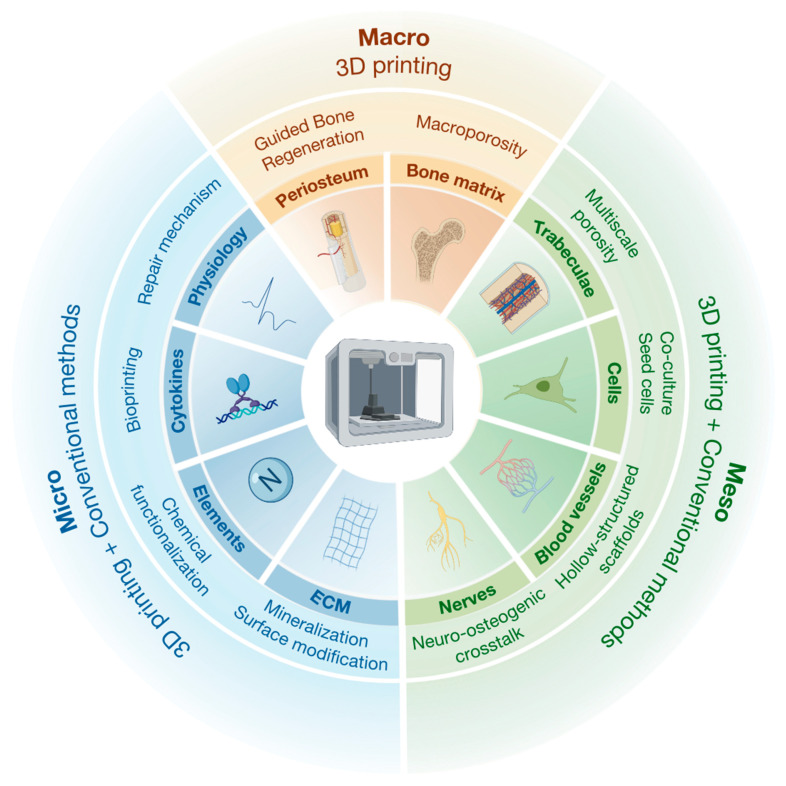
The hierarchical anatomical and physiological structures of bone inspire the multiscale design of scaffolds for BTE. The diagram illustrates three layers of information: (**i**) colors and outer bold labels indicate hierarchical levels—yellow for macro, green for meso, and blue for micro; (**ii**) inner icons and bold labels represent bone structures to be mimicked, such as periosteum, bone matrix, and trabeculae; (**iii**) middle-layer labels denote engineering strategies inspired by these structures, for example, guided bone regeneration (GBR) for periosteum and multiscale porous scaffolds for bone matrix and trabeculae.

To systematically translate multiscale biological inspirations into scaffold design, Figure 3 presents an integrated framework outlining the key structural features and regulatory elements across macro-, meso-, and microscale levels. This schematic emphasizes the synergistic application of 3D printing and traditional manufacturing techniques in bone tissue engineering, illustrating how distinct hierarchical cues—ranging from macroporosity and trabecular architecture to ECM-mimetic nanostructures—can be incorporated into scaffold construction to recapitulate native bone complexity.

## 5. Macroscale Construction Techniques and Strategies for BTE Scaffolds

### 5.1. Three-Dimensional Printing Technologies for Macroscale Structural Construction

**Figure 4:**
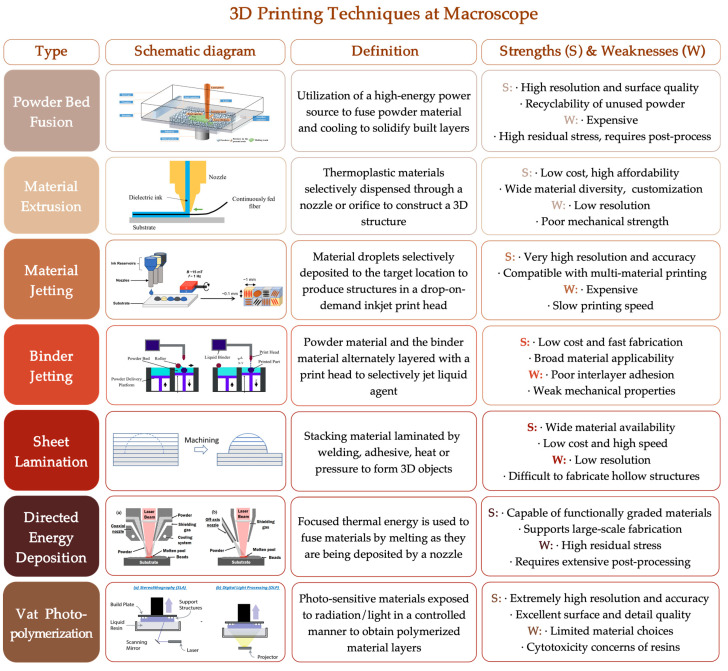
Macroscale 3D printing techniques for bone scaffold construction. The methods are categorized into Powder Bed Fusion [39,40,41] (Reprinted with permission from Ref. [40], Copyright 2020, Taylor & Francis), Material Extrusion [42,43,44] (Reprinted with permission from Ref. [42], Copyright 2024, Springer Nature), Material Jetting [45,46,47,48,49] (Adapted from Ref. [47] under the terms of CC BY), Binder Jetting [50,51] (Adapted with permission from Ref. [50], Copyright 2023, Elsevier), Sheet Lamination [52,53,54] (Adapted with permission from Ref. [53], Copyright 2022, Springer Nature), Directed Energy Deposition [55] (Reprinted from Ref. [55] under the terms of CC BY), Vat Photopolymerization [56] (Adapted from Ref. [56] under the terms of CC BY).

According to varied fundamental forming mechanisms, conventional 3D printing techniques can be broadly categorized into seven main types: powder bed fusion [39,40,41], material extrusion [42,43,44], material jetting [45,46,47,48,49], binder jetting [50,51], sheet lamination [52,53,54], directed energy deposition [55], and vat photopolymerization [56] (Figure 4). These approaches encompass a wide range of material systems—including metals, bioceramics, and polymers—and provide powerful fabrication tools for the macroscopic structural design of BTE scaffolds [57,58].

As tissue engineering and personalized medicine continue to advance rapidly, conventional 3D printing technologies are evolving toward higher-dimensional structural fabrication and functional integration, driving the emergence of a new generation of advanced additive manufacturing strategies. For instance, emerging technologies such as 3D bioprinting [59,60] and 4D printing [61,62] have transcended the static, layer-by-layer deposition paradigm of traditional methods by incorporating innovations such as temporal dynamics, stimulus responsiveness, and hierarchical structural construction. These approaches enable programmable shape transformation and multi-component co-deposition, offering significant advantages in multiscale structural control and biological functionality integration.

### 5.2. Strategies for Macroscale Structural Construction

**Figure 5:**
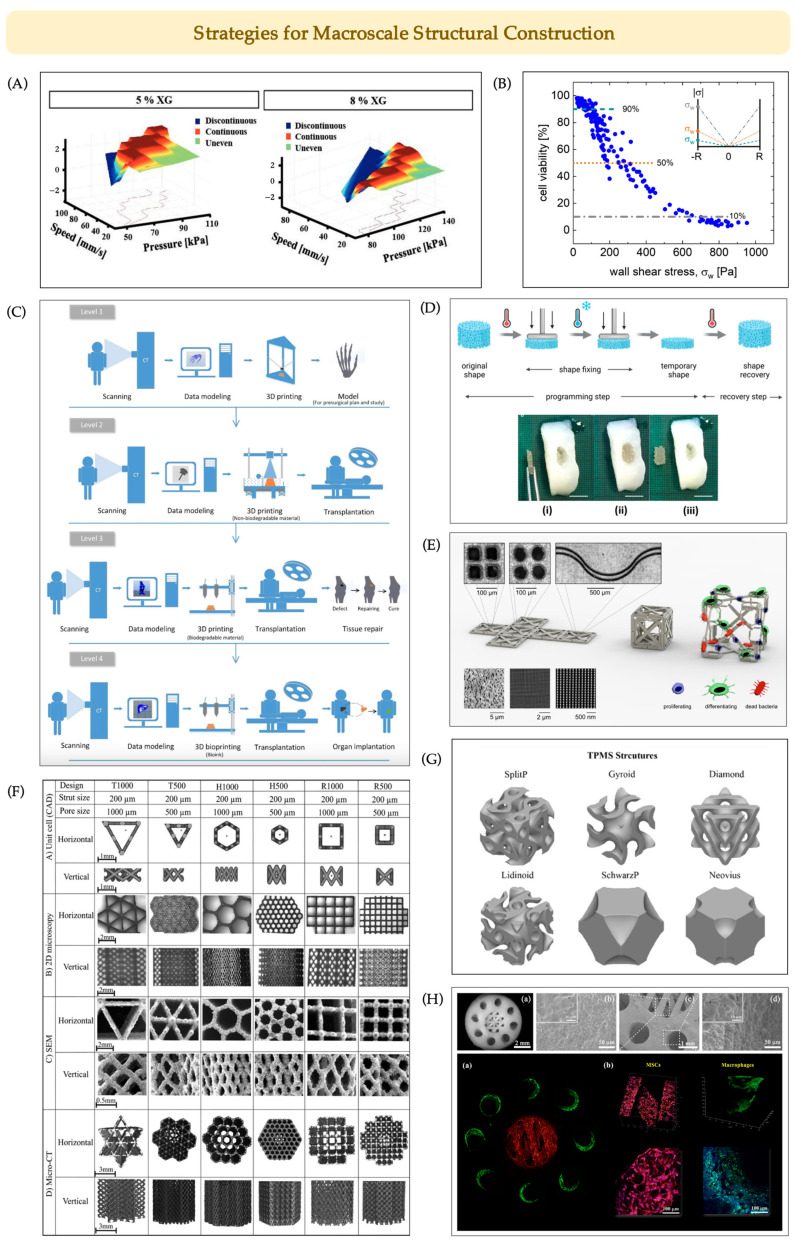
Macroscopic Structural Fabrication Techniques and Strategies for BTE Scaffolds. (**A**) Three-dimensional distribution plots with projections showing the classification of printing outcomes (discontinuous, continuous, uneven) for 5% and 8% xanthan gum (XG) bioinks under different pressures and printing speeds. Adapted with permission from Ref. [67], Copyright 2021, IOPScience. (**B**) Relationship between wall shear stress and cell viability during extrusion-based bioprinting. Reprinted from Ref. [69] under the terms of CC BY. (**C**) Four levels of 3D printing showing the progression of 3D printing in medicine. Reprinted with permission from Ref. [70], Copyright 2019, Elsevier. (**D**) Illustration of the thermally induced shape-memory effect, (**i**) Programmed temporary shape; (**ii**) Shape recovery in situ; (**iii)** Scaffold removed for evaluation. Adapted from Ref. [90] under the terms of CC BY. (**E**) Schematic diagram of foldable meta-implants with different surface-related nanopatterns. Reprinted from Ref. [73] under the terms of CC BY. (**F**) Representative scaffold design with different pore sizes, strut thicknesses, and pore shapes. Adapted with permission from Ref. [91], Copyright 2017, Elsevier. (**G**) Representative lattices of TMPS structures. Adapted with permission from Ref. [92], Copyright 2025, Springer Nature. (**H**) Haversian bone-mimicking bioceramic scaffolds and fluorescence microscopy images of the orderly distribution of MSCs and macrophages on the scaffolds. Adapted with permission from Ref. [93], Copyright 2021, Elsevier.

Additive manufacturing (AM) for BTE can be divided into pre-, intra-, and post-printing stages. In the pre-printing stage, polymeric materials with poor biocompatibility often require modification [63]. Among bioprinting modalities, extrusion-based bioprinting has become mainstream, owing to its compatibility with diverse materials (hydrogels, polymers, ceramics), ability to handle cell spheroids and high-viscosity inks, and its non-thermal nature that preserves bioactive factors. The rheological properties of bioinks critically determine printability, structural fidelity, and cell viability [64,65]. For example, tuning ionic crosslinking time alters shear-thinning behavior and cell activity [66], while shear-thinning xanthan gum methacrylate (XGMA) improves gelatin methacryloyl (GelMA) stability and post-printing viability (Figure 5A) [67]. However, excessive shear stress remains a major limitation: nozzle diameters below 150 μm or pressures above 40 psi significantly reduce immediate cell survival [68]. A recent predictive model by McCauley et al. [69] further integrates nozzle size, flow rate, rheology, and cellular mechanics, confirming that reduced nozzle radius, higher flow speed, and elevated viscosity all exacerbate cell damage (Figure 5B). These findings highlight nozzle geometry–pressure matching as a prerequisite for balancing fidelity with viability in extrusion-based bioprinting.

To meet the growing clinical demands for personalized and functional regeneration, 3D printing in BTE has evolved from basic presurgical modeling to fully functional, biomimetic, and even living implants (Figure 5C) [70]. This development trajectory, spanning from conventional additive manufacturing to 4D printing [71], which offers programmable deployment and defect-specific conformability (Figure 5D), highlights the urgent need for more adaptable and smart design approaches. In terms of external design, researchers have explored modular and other shape-adaptive strategies [72] to improve scaffold conformity to bone defects. For example, Zadpoor et al. [73] proposed a balloon inflation—triggered mechanism enabling polyhedral struts [74] or TPMS lattices [75] to expand from compact to load-bearing states, facilitating minimally invasive implantation (Figure 5E). The concept of “metallic clay” [76] also offers greater forming flexibility for reconstructive surgery. Internally, a wide variety of unit cell geometries (Figure 5F)—including diamond [77], octahedral [78], TPMS (Figure 5G) [79] and gradient designs, are now increasingly replaced by biomimetic morphologies such as the Haversian system (Figure 5H) [80,81], loofah [82,83], lotus root, and leaf [84]. Loofah-inspired scaffolds with coexisting macro- and micropores exhibit enhanced energy absorption and crushing resistance [83], while leaf-vein–like membranes promote osteoblast and fibroblast adhesion [85], supporting hard/soft tissue regeneration.

After printing, structural refinement is achieved by mechanical, chemical, or biological post-processing (e.g., support removal, polishing, solvent washing, sintering, bio-coatings) [86,87,88,89], which improve scaffold mechanics and bioactivity. Traditional methods such as freeze-drying and electrospinning are also integrated for meso-/microscale regulation, representing emerging directions for scaffold optimization.

## 6. Mesoscale Construction Techniques and Strategies for BTE Scaffolds

### 6.1. Three-Dimensional Printing Technologies for Mesoscale Structural Construction

**Figure 6:**
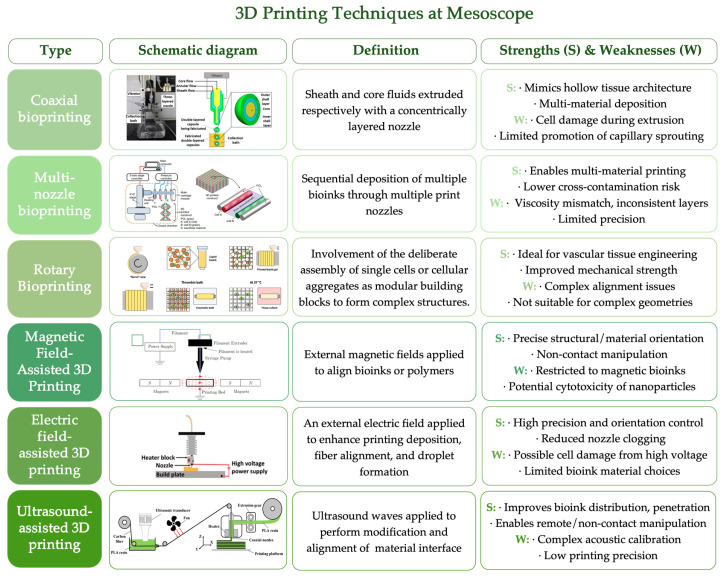
Mesoscale 3D printing techniques for bone scaffold construction. The methods are categorized into Coaxial Bioprinting [94,95] (Adapted with permission from Ref. [95], Copyright 2022, IOPScience), Multi-nozzle Bioprinting [96,97] (Adapted with permission from Ref. [96], Copyright 2021, John Wiley and Sons), Rotary Bioprinting [98] (Adapted with permission from Ref. [98], Copyright 2019, Elsevier), Magnetic Field-Assisted 3D Printing [47,99,100] (Reprinted with permission from Ref. [99], Copyright 2022, Elsevier), Electric field-assisted 3D printing [101,102,103] (Reprinted from Ref. [102] under the terms of the CC BY) and Ultrasound-assisted 3D printing [104,105,106] (Adapted with permission from Ref. [104], Copyright 2019, Elsevier).

Representative derived printing techniques for mesoscale construction include coaxial bioprinting [94,95], multi-nozzle bioprinting [96,97], and rotary bioprinting [98]. In parallel, external physical field-assisted approaches—such as magnetic field-assisted [47,99,100], electric field-assisted [101,102,103], and ultrasound-assisted [104,105,106] 3D printing—have emerged as promising strategies for regulating material alignment, cellular distribution, and directional growth during the fabrication process (Figure 6). These advanced methods expand the functionalization potential of scaffolds by enabling enhanced spatial control. While each technique offers unique advantages, they also present technical challenges and limitations in practical applications. Current printing resolution often struggles to precisely reproduce features in the sub-100 μm range, resulting in poorly defined pore boundaries or irregular geometries due to collapses and fusion of adjacent layer [107]. In particular, the fabrication of multi-material and multifunctional 3D-printed scaffolds (e.g., drug release, conductivity, vascularization) remains technically challenging and limited in practice [108], consequently conventional techniques such as freeze-drying, gas foaming, and electrospinning are often combined to achieve multifunctionality.

### 6.2. Strategies for Mesoscale Structural Construction

To accurately replicate the mesoscopic hierarchical architecture during 3D printing, researchers have developed a variety of strategies that can be broadly categorized into material-based and method-based approaches. On the material side, incorporating inorganic fillers—such as hydroxyapatite, β-tricalcium phosphate (β-TCP), or silica—into organic polymer matrices has been shown to broaden particle size distributions, improve surface energy and roughness, and enhance mineralization potential. These modifications facilitate stem cell adhesion and differentiation through organic–inorganic synergistic effects [109,110,111,112,113].

This review primarily focuses on the methodological dimension—namely, the integration of multiple fabrication techniques to construct scaffolds with coexisting macroporous and microporous architectures. In recent years, increasing attention has been directed toward designing multiscale hierarchical scaffolds that combine both pore types to optimize mechanical integrity and biological performance. In this strategy, scaffolds with pore sizes greater than 300 μm are considered capable of supporting cell migration and nutrient exchange [114], whereas micropores smaller than 10 μm enhance the scaffold’s osteoinductive capacity [115]. Collectively, multiscale scaffolds combining pores of different sizes promote improved osteointegration, bone regeneration, and expansion of the bone–scaffold interface compared to their single-scale counterparts [116].

Representative studies that integrate traditional manufacturing and 3D printing to construct mesoporous bone scaffolds are summarized in Table 1.

#### 6.2.1. Traditional Techniques for Mesoscale Pre-Processing in 3D Printing

**Figure 7:**
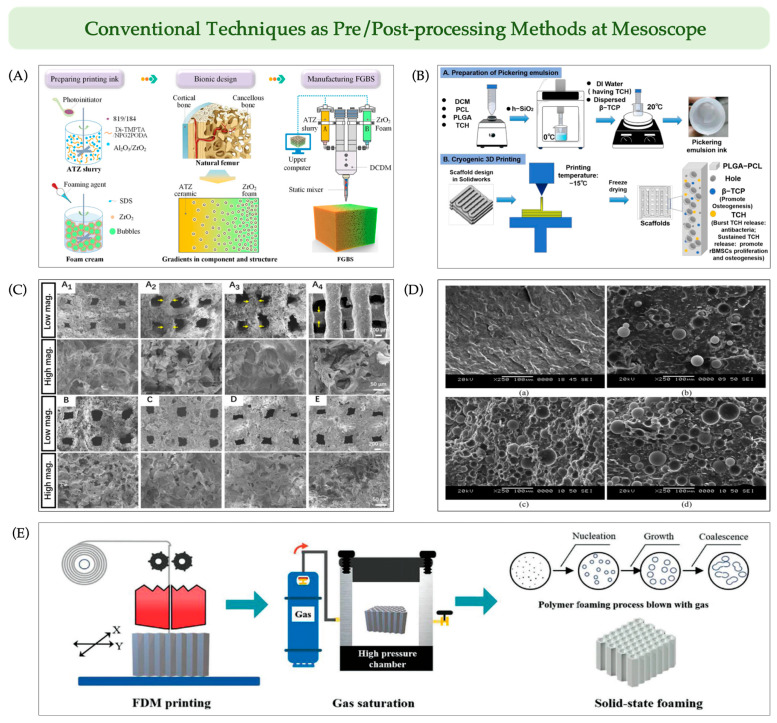
Integration of conventional mesoscale pre- and post-processing strategies into 3d printing of bone scaffolds. (**A**) Gradient scaffold fabricated by pre-foaming with supercritical CO_2_ followed by 3D printing. Reprinted with permission from Ref. [129], Copyright 2023, Elsevier. (**B**) Low-temperature micro-extrusion 3D printing based on Pickering emulsion inks combined with post-printing freeze-drying to form stable structures. Reprinted from Ref. [131] under the terms of CC BY. (**C**) SEM images of scaffolds fabricated using different Pickering emulsion inks (**A_1_**–**E**). Yellow arrows indicate strut thinning. Each pair shows top views at low (50×) and high (350×) magnification. Adapted from Ref. [131] under the terms of CC BY. (**D**) Cryofractured SEM images of HDPE-based filaments. (**a**–**d**) Filaments with 0, 20, 40, and 60 vol% cenospheres, respectively. Adapted with permission from Ref. [133], Copyright 2020, Elsevier. (**E**) Schematic diagram of foam fabrication by post-3D-printing foaming of printed structures. Reprinted with permission from Ref. [134], Copyright 2021, Elsevier.

In recent years, functionally graded bone scaffolds have garnered considerable attention due to their superior performance in interfacial integration and the synergistic modulation of mechanical and biological properties. To achieve stable interfacial bonding within continuous gradient architectures, traditional foaming templates have been introduced as a pre-processing strategy prior to 3D printing, assisting in the construction of functional inks [128]. For instance, one study employed a combination of alumina-toughened zirconia (ATZ) slurry and ZrO_2_ foam paste, using a static mixer to generate a compositional gradient, thereby enabling the simultaneous formation of structural and compositional gradients within a single printing step [129] (Figure 7A).

In addition, syntactic foams represent another effective pretreatment strategy. These foams incorporate hollow microspheres (e.g., glass or polymer microspheres) into polymers to create high-density composites that can be directly used in 3D printing. In work by Bonthu et al. [130] microsphere-reinforced printed constructs successfully retained the integrity of the microspheres during fabrication, resulting in closed-cell architectures with a porosity of 5–10%. Although this approach improved the tensile modulus, it also led to the formation of interlayer air gaps, which significantly reduced tensile strength, ductility, and toughness—highlighting the need for further optimization of pretreatment protocols to overcome such structural defects.

#### 6.2.2. Traditional Techniques for Mesoscale Post-Processing in 3D Printing

Recently, various post-processing techniques have been explored to refine scaffold architecture at the mesoscopic scale and improve functional performance after initial 3D printing fabrication. For instance, Ye et al. [131] employed a Pickering emulsion ink composed of dichloromethane (DCM), PLGA-PCL, tetracycline hydrochloride (TCH), and β-TCP for 3D printing, followed by freeze-drying of the 3D-printed scaffolds. This approach successfully yielded hierarchically porous scaffolds with a macroporous grid structure (average pore size 250.03 ± 75.88 μm) and interconnected micropores (average pore size 24.70 ± 15.56 μm). The resulting scaffolds not only exhibited good structural stability but also demonstrated excellent antibacterial activity and osteogenic potential, making them suitable for repairing infectious bone defects (Figure 7B,C).

Gas foaming represents another widely used post-processing strategy for micropore generation. In one study, Park et al. [132] subjected FDM-printed PLA scaffolds to a high-pressure CO_2_ environment, inducing nanoporous structures via physical foaming. By precisely tuning foaming parameters such as pressure and temperature, the pore size and porosity could be effectively controlled. Similarly, cryofractured SEM observations of high density polyethylene (HDPE)-based filaments containing increasing vol% of cenospheres (0–60 vol%) (Figure 7D) clearly demonstrate the feasibility of gas-foaming–induced microstructural modulation in polymer-based scaffolds [133].

In addition, intermittent foaming using dry ice (solid CO_2_) has also been explored as a post-processing strategy (Figure 7E). Hu et al. [134] reported that microporous foaming of thermoplastic polyurethane (TPU) honeycomb structures increased energy absorption efficiency from 0.32 to 0.38 to 0.40, while significantly enhancing elasticity and recovery capacity. However, such lab-scale foaming techniques face limitations in scalability. Therefore, the efficient fabrication of industrial-grade foamed structures remains a key challenge and a critical direction for the integration of 3D printing with conventional manufacturing technologies.

#### 6.2.3. Mesoscale-Oriented Integration of Traditional and 3D Printing Techniques

##### 3D Printing + Freeze-Drying

**Figure 8:**
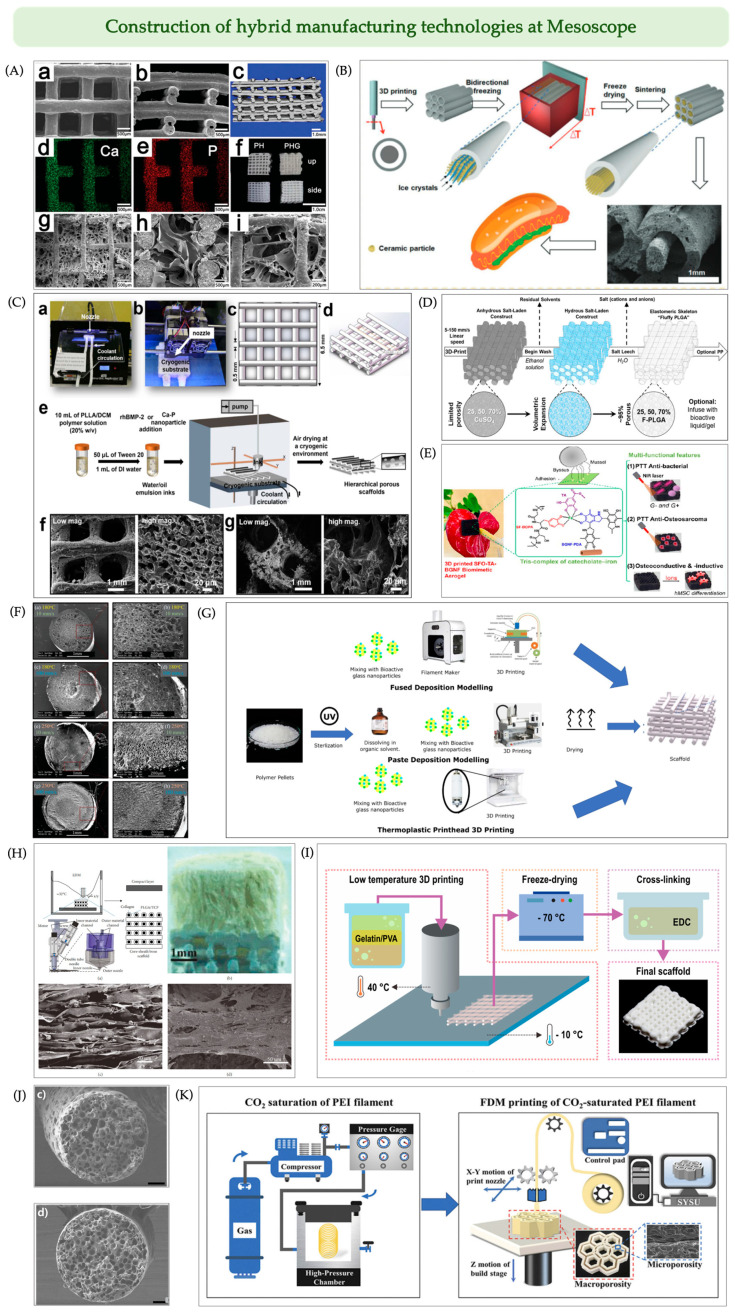
Construction of hybrid manufacturing technologies at the mesoscale for advanced scaffold fabrication. (**A**) PLGA/HA scaffold shown in SEM (**a**,**b**) and micro-CT (**c**); the distribution of Ca (**d**) and P elements (**e**) in the PLGA/HA scaffold; macro photograph of scaffolds (**f**); freeze-dried internal gelatin network (**g**–**i**). Adapted with permission from Ref. [119], Copyright 2021, Royal Society of Chemistry. (**B**) The schemata of preparation of hot dog-like scaffolds, combining 3D printing and bidirectional freezing. Adapted from Ref. [120] under the terms of CC BY. (**C**) Cryogenic 3D printing of hierarchical porous scaffolds. (**a**,**b**) Modified printer setup; (**c**,**d**) CAD model; (**e**) Schematic of cryogenic printing process; (**f**,**g**) SEM images. Reprinted with permission from Ref. [137], Copyright 2017, IOPScience. (**D**) Schematic diagram of multifunctional scaffolds with dual-tunable porous structures fabricated by combining 3D printing and salt leaching. Adapted with permission from Ref. [121], Copyright 2018, Elsevier. (**E**) Multifunctional composite hydrogels processed into black, porous, and hierarchically structured composite aerogel scaffolds via 3D printing followed by supercritical drying. Reprinted with permission from Ref. [138], Copyright 2024, American Chemical Society. (**F**) SEM images depicting the cellular structure of the filaments at extreme printing temperatures (**a**–**d**: 180 °C and **e**–**h**: 250 °C) and speeds (**a**–**f**: 10 mm s^−1^ and **c**–**h**: 100 mm s^−1^). Adapted with permission from Ref. [139], Copyright 2017, Wiley. (**G**) Workflow for different strategies for extrusion-based 3D printing of thermoplastic polymers-bioactive glass scaffolds. Reprinted from Ref. [140] under the terms of CC BY. (**H**) Oriented cartilage ECM-derived scaffolds were fabricated by an improved TIPS process and low-temperature 3D deposition technology. Adapted with permission from Ref. [141], Copyright 2017, IOPScience. (**I**) Fabrication process of low-temperature deposition manufacturing (LTDM). Reprinted with permission from Ref. [142], Copyright 2018, Elsevier. (**J**) Cross-sectional SEM images of mono-filaments with different chain extender ratios (**c** = 1.0 wt%, **d** = 1.5 wt%) after gas foaming. Adapted with permission from Ref. [126], Copyright 2020, Elsevier. (**K**) Schematic of FDM printing process of the CO_2_-saturated PEI filament for gas foaming post-processing. Reprinted with permission from Ref. [143], Copyright 2020, Elsevier.

Freeze-drying, a technique performed under low temperature and pressure via ice crystal sublimation and water desorption, is widely employed to generate porous structures. When combined with 3D printing, it enables precise modulation of the scaffold’s mesoscale architecture, allowing for the fabrication of biomimetic scaffolds with hierarchical and layered pore structures [135]. The most direct approach involves the alternating stacking of printed and frozen layers to form multilevel channel networks. For instance, the integration of a 3D-printed PLGA/n-HA scaffold with an interconnected freeze-dried gelatin network forms distinct primary and secondary channels, effectively promoting cell infiltration and tissue integration [119] (Figure 8A).

To further enhance biological functionality, microporosity must be integrated into the 3D-printed layered porous constructs to achieve interconnected, hierarchical porosity. This requires printable “inks” with both extrusion stability and freeze-responsive behavior, presenting a major challenge in merging freeze-drying with AM. Song et al. [118] developed a low-temperature extrusion system, where a liquid nitrogen cooling plate beneath the direct ink writing (DIW) printing platform enables in situ directional freezing of viscous HA slurries during extrusion. The resulting freeze-dried scaffolds exhibited vertically aligned channels and interconnected micropores, significantly enhancing osteogenic potential. Similarly, Jung et al. [136] fabricated macro-/microporous HA scaffolds seeded with osteoblast-like cells, demonstrating superior cell guidance and bone regeneration compared to conventional freeze-cast-only constructs.

With the emergence of bidirectional freezing and electro-assisted freeze-drying, the structural stability and 3D pore orientation of scaffolds have further improved, and these techniques have now been successfully integrated into the 3D printing workflow. For example, combining DIW with bidirectional freeze-casting yielded a “hot-dog-like” scaffold comprising hollow ceramic tubes (~1 mm in diameter, simulating “buns”) encasing ceramic rods (~500 µm, simulating “sausages”), which enhanced rat bone marrow mesenchymal stem cell (rBMSC) delivery and bone formation (Figure 8B) [120].

Emulsion-based inks, particularly high internal phase emulsions (HIPEs), have demonstrated unique advantages in fabricating microporous scaffolds. When used as printable inks, the aqueous droplets in HIPEs serve as sacrificial templates that, upon polymerization and drying, generate ECM-mimicking microporous structures with high pore-forming efficiency [144]. Moreover, low-temperature printing offers a favorable environment for preserving biological activity, making it ideal for scaffolds requiring controlled release of proteins, enzymes, or drugs. Emulsion inks incorporating recombinant human bone morphogenetic protein-2 (rhBMP-2) and Ca–P nanoparticles, when printed on cryogenic platforms and freeze-dried, form layered microporous structures with controlled Ca^2+^ and growth factor release, promoting human bone marrow stromal cell (hBMSC) proliferation and osteogenic differentiation [137] (Figure 8C). Similarly, low-temperature direct writing (LTDW) has been applied in the fabrication of LiFePO_4_ (LFP) electrodes, demonstrating its versatility across both biomedical and energy storage domains.

##### 3D Printing + Particulate Leaching/Solvent Casting

Particle leaching/solvent casting refers to a process in which soluble salt particles (e.g., NaCl or CuSO_4_) are mixed with a polymer solution. After solvent evaporation, a solid composite is formed, followed by immersion in an aqueous phase to remove the salt, thereby generating microporous structures. The resulting pore morphology and porosity are entirely dictated by the size, shape, and distribution of the chosen template particles, offering precise control over microstructure formation [145]. By pre-mixing salt templates into printable inks, researchers have developed a “salt-printing” approach that enables the simultaneous fabrication of macroscale scaffold architecture and embedded microporosity. A representative study by Jakus et al. [121] demonstrated this concept (Figure 8D): copper sulfate (CuSO_4_) particles were incorporated into poly(lactic-co-glycolic acid) (PLGA)-based printing inks, yielding multifunctional scaffolds with dual-scale porosity. The macroscopic structure was shaped via 3D printing, while post-printing aqueous leaching of the salt particles generated controlled micropores. These scaffolds exhibited excellent mechanical performance, bioactivity, and multi-material compatibility, while maintaining simplicity, low cost, and tunability.

Despite these advantages, notable limitations persist. Complete removal of salt particles often requires multiple leaching steps, prolonging the fabrication process. Additionally, the use of organic solvents to dissolve polymers may lead to residual solvent contamination, raising concerns over biosafety and potential cytotoxicity [146].

##### 3D Printing + Sol—Gel Self-Assembly

Sol–gel self-assembly involves the hydrolysis and polycondensation of precursors to form a sol, which gradually transitions into a gel network with tunable pore structures. Upon drying and thermal treatment, a porous controllable structure is obtained at a low processing temperature [147,148]. Among sol–gel-derived materials, aerogels and bioactive glasses are the most widely applied. However, scaffolds prepared solely by sol–gel techniques typically suffer from poor mechanical properties [149,150,151], which can be effectively overcome by integrating 3D printing to produce mechanically robust, customized scaffolds.

Aerogels, as representative sol–gel products, are nanoporous materials formed via sol–gel transition or molecular self-assembly and dried using supercritical CO_2_ [152,153]. Their integration with 3D printing has been explored extensively. For instance, Maleki et al. [154] developed a silica–silk hybrid aerogel with hierarchical porosity and high mechanical strength via unidirectional freeze-casting and supercritical drying. Another study [123] introduced magnetic responsiveness by embedding superparamagnetic iron oxide nanoparticles (SPIONs) into 3D-printed aerogels, achieving remote actuation while retaining printing precision. In another example, a biomimetic aerogel composed of silk fibroin oxide, tannic acid, and bioactive glass fibers mimicked mussel adhesive mechanisms. Through dynamic coordination and supercritical drying, this construct yielded a porous, layered aerogel scaffold with self-healing properties and enhanced structural complexity [140] (Figure 8E,F).

Typically, bioactive glasses can be classified into silicate (SiO_2_), phosphate (P_2_O_5_), and borate (B_2_O_3_) systems, and can be fabricated in the form of porous scaffolds. These materials stimulate bone cell adhesion and proliferation and rapidly integrate with native bone tissue [155]. Their combination with 3D printing typically follows two main strategies [142] (Figure 8G). The first involves mixing bioactive glass particles with thermoplastics to form pastes, which are extruded via FDM and followed by solvent evaporation. However, the glass particles synthesized by traditional high-temperature methods are usually micron-sized and poorly compatible with extrusion-based printing. To address this and increase specific surface area, researchers have employed sol–gel (Stöber) synthesis to prepare mesoporous bioactive glass [156]. In this process, by adjusting the ammonium hydroxide volume and quantity of calcium nitrate, the particle size can be finely tuned for improved compatibility with 3D printing systems [156].

The second approach involves laser-assisted 3D printing using thermosetting polymer matrices. For example, poly(propylene fumarate) (PPF), although not inherently osteoconductive or osteoinductive, demonstrates significantly enhanced osteogenic potential when combined with bioactive glass [157].

More recently, efforts have focused on developing bioactive glass-based composites with bioactive components, including cells, genes, and growth factors, to further enhance regenerative potential [158]. Such composites are promising as bioinks for extrusion-based 3D printing. For instance, a multifunctional nanocomposite bioink was developed by integrating amine-functionalized Cu-doped mesoporous bioactive glass nanoparticles into an oxidized alginate–gelatin hydrogel, yielding favorable rheology, shape fidelity, and high cell viability (>90%), which represents a suitable approach for developing a new generation of bioinks incorporating bioactive fillers [159].

##### 3D Printing + Thermal-Induced Phase Separation

Thermally induced phase separation (TIPS) is a classical and facile method for fabricating porous materials, allowing precise control over pore size, geometry, and interconnectivity by tuning parameters such as cooling rate, polymer concentration, crystallinity, and ceramic content [160]. TIPS can be seamlessly integrated with 3D printing technologies to expand its potential applications in BTE [124]. Yousefi et al. embedded porous polyethylene glycol (PEG) structures into a PLGA-based composite system and removed the PEG phase via aqueous extraction, resulting in a hierarchical scaffold with combined macro- and mesoporosity, providing an ideal microenvironment for bone regeneration [124].

Inspired by such strategies, researchers have proposed low-temperature deposition manufacturing (LTDM), which integrates extrusion-based 3D printing with the TIPS process to enable the co-construction of macro-, meso-, and microscale porosities. For example, Ma et al. introduced magnesium into a PLGA/β- tricalcium phosphate (β-TCP) matrix and employed rapid cryogenic molding to fabricate functionalized scaffolds with inherent antibacterial properties [161]. Furthermore, by modifying the printhead, it is possible to achieve multi-material co-printing and core–shell architecture fabrication simultaneously. Zhang et al. [143] developed a coaxial nozzle capable of concurrently depositing a PLGA/TCP composite (core) and collagen (shell), which was then combined with TIPS-derived oriented cartilage ECM scaffolds to construct biomimetic osteochondral scaffolds with gradient interfaces (Figure 8H). Another LTDM approach involves direct extrusion of precursor solutions onto cryogenic substrates, enabling instant solidification and shape retention. Kim et al. [144] demonstrated gelatin/PVA scaffolds fabricated via this method with tunable physical and biological properties (Figure 8I), highlighting their potential for both soft and hard tissue regeneration.

Despite the outstanding morphological tunability of these techniques, their biomedical applications are still constrained by certain limitations, such as the layer height restrictions inherent to freeze casting and the potential cytotoxicity associated with organic solvents. To address these issues, researchers have been exploring safer phase separation strategies. For instance, Dong et al. [162] introduced a polymerization-induced phase separation (PIPS) mechanism into DLP printing to avoid solvent-related toxicity. In this method, photoinitiated polymerization drives phase separation during printing, enabling hierarchical pore formation from the nanoscale to the millimeter scale. This approach significantly enhances the scaffold’s specific surface area, biocompatibility, and molecular adsorption capabilities.

##### 3D Printing + Gas Foaming

Gas foaming involves the introduction of blowing agents (e.g., CrNx, TiH_2_, carbonates) or inert gases (e.g., N_2_, CO_2_) to induce bubble formation within a polymer matrix, generating high-porosity structures without the use of organic solvents [132]. However, conventional foaming methods often lack control over pore morphology and interconnectivity, limiting their standalone applications in tissue engineering. When integrated with 3D printing—especially FDM—gas foaming can enable the synergistic construction of macro- and microstructures, effectively compensating for the poor microstructural control of FDM technology. Moreover, the resulting microporous architecture can be finely tuned by adjusting printing parameters such as temperature and speed [141].

Recently, in situ foam printing technologies have attracted increasing attention. Zhang et al. [163] combined 3D fused filament fabrication (FFF) with supercritical microcellular foaming, achieving a uniform polymer matrix using physical blowing agents. Choi WJ et al. [126] incorporated chemical foaming agents (CFAs) such as azodicarbonamide into PLA-based filaments (Figure 8J), enabling one-step fabrication of dual-porosity scaffolds with high design flexibility and promising in vitro biological performance. The process allows tunability through chain extension reactions that adjust the rheological behavior of PLA.

Beyond conventional blowing agents, thermally expandable microspheres (TEMs) have also been utilized for in situ foaming during 3D printing [164,165]. These microspheres provide a more homogeneous porous architecture during extrusion or injection molding. For example, Andersson et al. employed PLA and a TEM–ethylene-vinyl acetate masterbatch to generate an in situ foamed structure via FFF printing [166]. Despite such advances, integrating gas foaming directly into 3D printing still poses challenges. Notably, non-uniform bubble expansion can lead to the formation of a dense outer skin layer, impairing pore interconnectivity and hindering cell infiltration.

To address this issue, Li et al. [145] reported a reliable strategy by impregnating CO_2_ into polyetherimide and PLA filaments to create layered porous scaffolds, currently among the most effective combinations of gas foaming with 3D printing. Similarly, Song et al. [127] developed a process (Figure 8K) in which PLA/PVA composites saturated with supercritical CO_2_ and containing a leachable PVA phase were printed using FDM to form scaffolds with macroscopic channels (300–700 μm). Subsequent thermal water bath foaming generated interconnected micropores (2–10 μm), while PVA was simultaneously leached out, yielding a multiscale porous structure with excellent interconnectivity.

Interestingly, gas foaming can also be achieved chemically under mild conditions, using degradable metals such as magnesium. For instance, calcium phosphate cement was infiltrated into additively manufactured WE43 magnesium alloy scaffolds (with ~75% porosity). Upon hydrothermal treatment, a reaction between magnesium and water released H_2_ gas, forming a porous architecture. This process yielded biodegradable Mg/hydroxyapatite interpenetrating composites, where the released Mg^2+^ ions promoted osteoblast differentiation and new bone formation [167].

## 7. Microscale Construction Techniques and Strategies for BTE Scaffolds

### 7.1. Three-Dimensional Printing Technologies for Microscale Structural Construction

**Figure 9:**
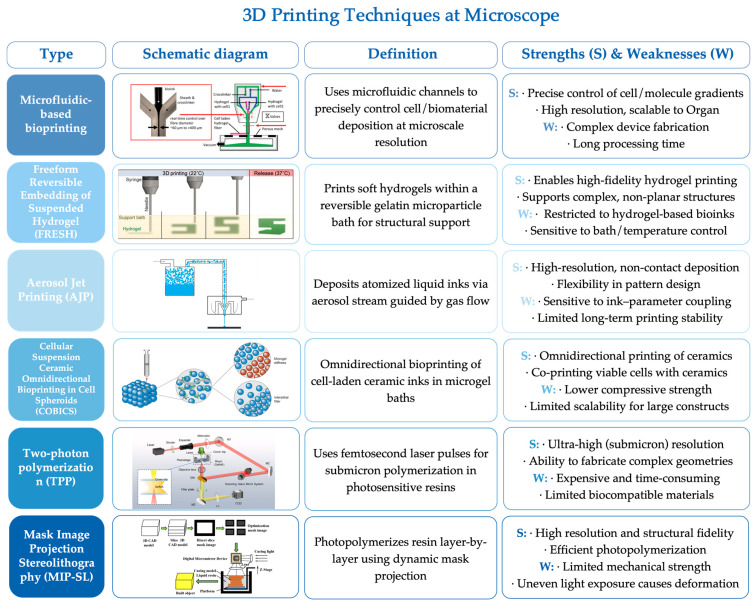
Microscale 3D printing techniques for bone scaffold construction. The methods are categorized into Microfluidic-based Bioprinting [169,170,171] (Adapted with permission from Ref. [169], Copyright 2019, John Wiley and Sons), FRESH [172,173,174] (Adapted from Ref. [174] under the terms of CC BY), AJP [175,176] (Adapted from Ref. [175] under the terms of CC BY), COBICS [177,178,179] (Adapted with permission from Ref. [179], Copyright 2023, Elsevier), TPP [180,181,182,183] (Reprinted from Ref. [182] under the terms of CC BY) and μMIP-SL [184,185] (Adapted with permission from Ref. [184] Copyright 2019, Springer Nature).

Recent evidence indicates that the nanotopography of biomaterials itself plays a critical role in regulating stem cell fate through mechanotransduction, which is crucial for elucidating the relationship between nanoscale structures, cell adhesion, and osteogenic differentiation [168]. The demand for precisely controlled nanotopographies has driven the emergence of a variety of high-resolution additive manufacturing technologies, opening new avenues for the direct fabrication of scaffolds at the micro- and nanoscale. These advanced techniques can be broadly categorized into two groups. The first category comprises extrusion-based methods, including microfluidic-based bioprinting [169,170,171], Freeform Reversible Embedding of Suspended Hydrogels (FRESH) [172,173,174], Aerosol Jet Printing (AJP) [175,176], and Cellular Suspension Ceramic Omnidirectional Bioprinting in Cell Spheroids (COBICS) [177,178,179]. The second category encompasses high-precision photopolymerization-based technologies, such as Two-Photon Polymerization (TPP) [180,181,182,183] and Micro Mask-Image Projection Stereolithography (μMIP-SL) [184,185] (Figure 9). While high-resolution printing techniques are often constrained by scale limitations, Greer et al. [186] developed a large-volume nanoscale 3D printing strategy by fusing nanoimprinting with digital light projection, which enables the fabrication of centimeter-scale constructs with nanoscale features at a truly rapid rate (~120 mm/h), and holds the world record for maintaining nanoscale surface fidelity. These state-of-the-art fabrication strategies—featuring sub-100 nm resolution—demonstrate exceptional potential in the precise reproduction of bone-mimetic microarchitectures and are steadily advancing the field of tissue engineering scaffolds toward greater precision and functionality.

### 7.2. Strategies for Microscale Structural Construction

#### 7.2.1. Traditional Techniques for Microscale Pre-Processing in 3D Printing

Surface micro-modification techniques represent a commonly employed approach for fabricating microscale features, typically utilized to impart critical biophysical cues onto scaffold surfaces. These modifications are generally implemented as post-processing steps and include physical-mechanical methods (e.g., sandblasting, laser texturing), chemical–electrochemical methods (e.g., acid etching, alkali treatment, anodic oxidation, micro-arc oxidation), and bio-coating approaches. However, in specific scenarios, particularly in chemical–electrochemical treatments, such techniques can also function as pre-processing strategies.

**Figure 10:**
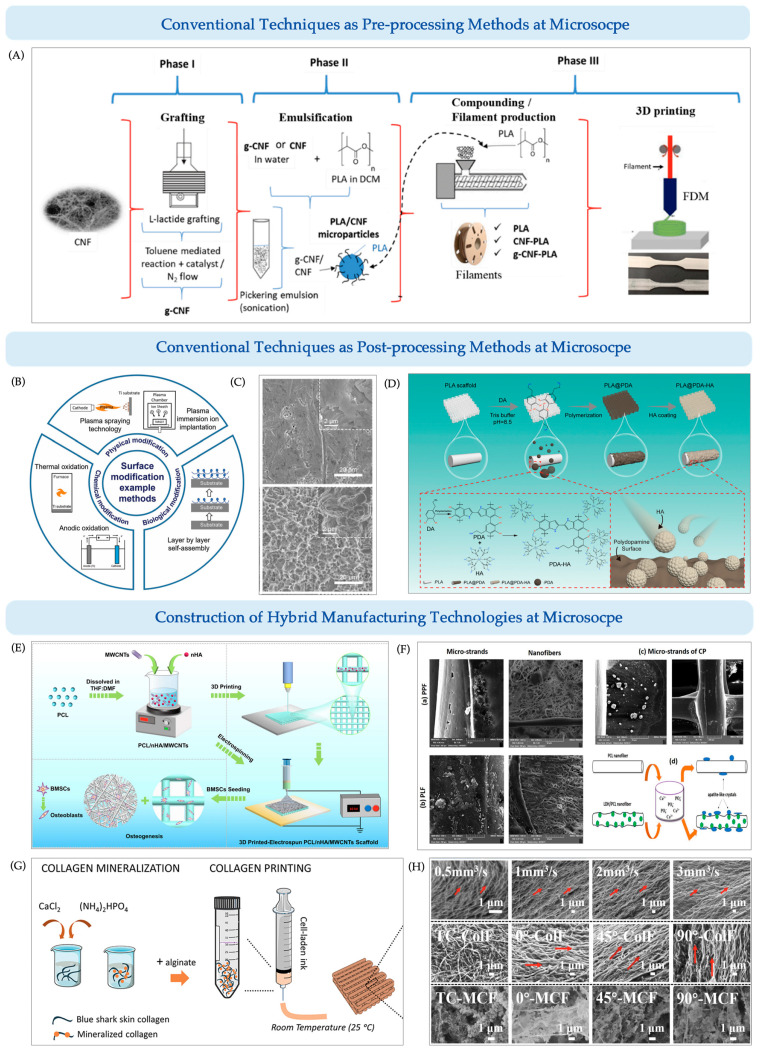
Microscopic Structural Fabrication Techniques and Strategies for BTE Scaffolds. Pre-processing methods: (**A**) PLA grafted with cellulose nano-fibrillated fibers (CNF) for 3D printing with enhanced tensile and thermo-mechanical properties. Reprinted with permission from Ref. [187], Copyright 2023, Elsevier. Post-processing methods: (**B**) Schematic diagram of representative surface modification methods. Reprinted from Ref. [188] under the terms of CC BY. (**C**) Nanofiber structure prepared on a titanium surface by alkali-hydrothermal treatment. Adapted from Ref. [189] under the terms of CC BY. (**D**) Schematic illustration of PDA-HA coating process of 3D-printed PLA scaffolds. Reprinted from Ref. [190] under the terms of CC BY. Construction of hybrid manufacturing technologies at the microscale for advanced scaffold fabrication: (**E**) Schematic illustration of preparation of 3D printed-electrospun PCL/nHA/multi-walled carbon nanotubes (MWCNTs) scaffold for bone regeneration. Reprinted from Ref. [191] under the terms of CC BY. (**F**) SEM images of 3D-printed grids and electrospun nanofibers. Adapted with permission from Ref. [192], Copyright 2023, Springer Nature. (**G**) Schematic illustration of bioprinting using collagen mineralized by alternate soaking as a bioink material. Adapted with permission from Ref. [193], Copyright 2020, American Chemical Society. (**H**) Micromorphology of collagen fibers and mineralized collagen fibers in 3D-printed scaffolds under varying extrusion rates and printing angles. Adapted with permission from Ref. [194] Copyright 2024, IOPScience.

For instance, in inkjet or stereolithography (SLA) printing, if poor interfacial adhesion is observed between the printing material and the substrate, pre-treatment of the substrate using plasma activation, chemical modification, or silanization can be applied to enhance surface hydrophilicity and interfacial bonding. This is particularly crucial for applications such as microfluidic chips, where polydimethylsiloxane (PDMS) substrates are commonly pre-treated to improve hydrogel attachment. Alternatively, for thermoplastic polymers like PLA, surface chemical modification of PLA granules prior to extrusion can improve the thermal and mechanical properties of the final 3D printed constructs (Figure 10A) [187].

#### 7.2.2. Traditional Techniques for Microscale Post-Processing in 3D Printing

For metallic scaffolds, especially those composed of titanium, magnesium, and their alloys, commonly used surface modification techniques include both physical-mechanical and chemical–electrochemical approaches (Figure 10B,C). These methods enable the formation of nanotubes, nanoneedles, nanowires, or micropores on the surface, significantly enhancing cell adhesion, bioactivity, and osseointegration capacity [195]. For instance, anodic oxidation produces well-ordered titanium nanotube arrays that facilitate osteoblast adhesion and elongation [196], while micro-arc oxidation (MAO) can incorporate bioactive elements such as calcium and phosphorus into the oxide layer, further improving osteogenic potential [197].

For polymeric scaffolds, in addition to surface treatments such as oxygen plasma and chemical etching, biofunctionalization using bioceramics, proteins, or living cells is frequently employed to enhance bioactivity. For example, γ-irradiation on PCL scaffolds has been shown to improve cell adhesion and growth factor release [198]. For inherently bioinert materials like PEEK, sulfonation treatment can introduce microporous structures and, when combined with bioactive coatings, significantly promote cell adhesion and differentiation [199]. Similarly, a polydopamine-hydroxyapatite (PDA-HA) coating fabricated on 3D-printed PLA has been reported to improve scaffold hydrophilicity and in vitro calcium release performance [190] (Figure 10D).

#### 7.2.3. Microscale-Oriented Integration of Traditional and 3D Printing Techniques

In addition to scaffold fabrication, the precise control of collagen assembly and biomimetic mineralization remains a critical challenge in BTE. Recent efforts have combined electrospinning, electrocompaction, and other nanoscale assembly techniques with in vitro mineralization to achieve controlled inorganic deposition onto collagen matrices. These strategies have enabled the development of structurally and functionally biomimetic scaffolds and laid the groundwork for high-resolution mineralized tissue engineering.

A summary of representative studies employing hybrid strategies that integrate traditional techniques with 3D printing for the construction of microporous bone scaffolds is presented in the following table (Table 2).

##### 3D Printing + Electrospinning

Electrospinning, a well-established nanomanufacturing technology, can efficiently produce polymer fibers at the micro- to nanoscale, making it ideal for replicating the topological features of natural bone ECM. The most common integration with 3D printing is via functional layer attachment, where the printing and electrospinning steps are performed separately and alternately on the same substrate to achieve layered structures. For example, Cao et al. [191] developed a composite scaffold by printing a PCL/nHA/MWCNTs framework and reinforcing it with electrospun nanofibers of the same composition (Figure 10E), resulting in a dense, well-integrated nanofiber layer that significantly improved cell adhesion and proliferation. However, challenges such as fiber misalignment and weak interfacial bonding persist, particularly limiting the mechanical performance in load-bearing applications [191].

A more robust strategy is hybrid fabrication via bonding, where electrospun membranes are affixed to printed scaffolds using adhesives. Belgheisi et al. [192] created such a hybrid scaffold by sandwiching PCL or PCL/LDH electrospun mats between two printed PCL grids, bonded using a 15% PCL solution in dichloromethane/dimethylformamide (2:1 *v*/*v*), applied at 20 droplets per cm^2^ to ensure firm adhesion (Figure 10F).

More advanced integration strategies include coaxial printing and melt electrowriting (MEW)—the most representative and frontier technologies at the intersection of 3D printing and electrospinning [202,203]. Coaxial printing enables simultaneous co-deposition of microscale filaments and nanofibers using a coaxial nozzle setup, allowing spatial control over material and architecture [204]. Electrospinning modules are attached externally to the printing head, enabling synchronized or alternating deposition of nanofibers during scaffold fabrication. MEW, by contrast, emphasizes fiber-level path precision and structural order. It integrates near-field electrospinning (NFES) with FDM, enabling layer-by-layer deposition of micro/nanofibers under CAD control to form highly ordered porous architectures [205,206]. For instance, Park et al. [207] used combined MEW and FDM to fabricate ECM-mimetic hierarchical scaffolds, significantly enhancing cell attachment and proliferation.

##### 3D Printing + In Vitro Mineralization

To date, the development of hard tissue substitutes has primarily relied on collagen templates followed by in vitro mineralization, including simple chemical co-precipitation and more biomimetic approaches such as simulated body fluid (SBF) [208] and polymer-induced liquid precursor (PILP) [209] methods. However, these strategies are mostly limited to casted or freeze-dried collagen matrices and offer limited spatial control over hierarchical collagen organization.

The emergence of advanced 3D printing technologies, especially FRESH printing, now allows for the spatiotemporal control of collagen alignment and scaffold microarchitecture by precisely tuning temperature, pH, and printing parameters. This has enabled the construction of highly ordered and dense collagen networks, which are otherwise difficult to achieve through conventional means. On this basis, the incorporation of SBF [210] or PILP [211] components as mineralization inducers enables deep intrafibrillar mineralization, offering unprecedented opportunities to fabricate bone-mimetic functional scaffolds.

Alternate soaking is another commonly used method for inducing mineralization on porous scaffolds or collagen-based matrices. Compared with immersion in static mineralizing solutions, this approach is faster, milder, and suitable for the gradual internal mineralization of complex 3D printed constructs [212,213]. For instance, Diogo et al. [193] employed alternate soaking to achieve in situ hydroxyapatite mineralization of blue shark collagen, which was subsequently blended with alginate to create printable, cell-laden hydrogels without the need for toxic crosslinkers (Figure 10G). Zhou et al. [194] demonstrated that shear forces during 3D printing can align collagen fibers directionally, producing mineralized oriented collagen fiber (ColF) scaffolds with significantly enhanced mechanical and osteogenic properties (Figure 10H). This strategy highlights the synergistic potential of collagen alignment and post-printing mineralization, offering a promising route toward engineered collagen scaffolds.

## 8. The Final Step of BTE Scaffolds—Towards Clinical Application

Irregular bone defects pose complex three-dimensional geometries and boundary conditions, making treatment costly and often unsatisfactory [214]. Despite decades of research, only a few BTE strategies have reached clinical approval, mostly as single-component products [215]. Currently, several 3D-printed biomaterials and scaffolds are commercially available for indications such as long bone defects, spinal fusion, periarticular defects, and cranio-maxillofacial reconstruction, with varying levels of clinical validation (Table 3).

### 8.1. In Vivo Evidence and Performance

**Figure 11:**
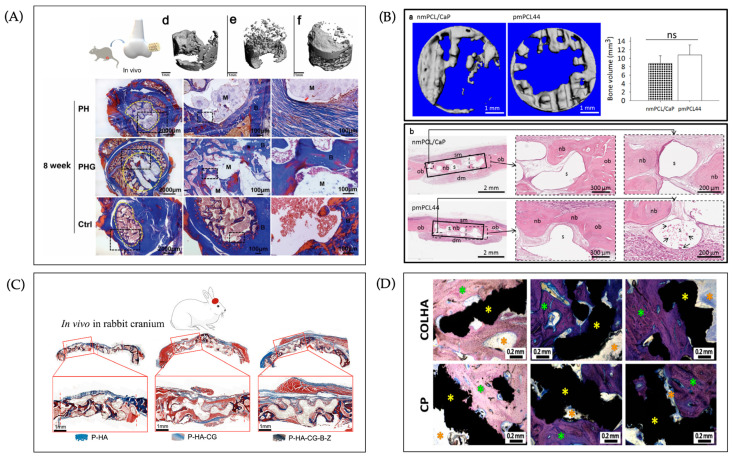
Representative in vivo evaluations of hybrid 3D printing and conventional techniques for bone tissue engineering. (**A**) In vivo implantation procedure, micro-CT reconstruction of defect areas (**d**–**f**), and Masson’s trichrome–stained sections at 8 weeks. Adapted from Ref. [119], Copyright 2021, Royal Society of Chemistry. (**B**) Micro-CT (**a**) and H&E-stained sections (**b**) of rat calvarial defects at 8 weeks. ob: old bone; nb: new bone; s: scaffold; sm: skin-side membrane; dm: dura-side membrane; black arrow: infiltrated extracellular materials. Adapted from Ref. [122], Copyright 2020, Elsevier. (**C**) Masson’s trichrome staining of rabbit calvarial defects. Adapted from Ref. [221], Copyright 2020, American Chemical Society. (**D**) Pig femur histological images (Giemsa’s azur–eosin–methylene blue staining) of printed titanium implants with or without collagen/hydroxyapatite (COLHA) layers. Green stars: bone tissue; orange stars: connective tissue; yellow stars: implants. Adapted from Ref. [222] under the terms of CC BY.

Most bone tissue engineering (BTE) strategies remain at preclinical or early translational stages, hindered by conflicts between mechanical strength and host integration [216], scalability and reproducibility issues [217], high manufacturing costs [218], and stringent regulatory requirements for multi-component systems [215]. Rodent calvarial models are widely used for early screening [219], but fail to replicate human load-bearing conditions, whereas large animals (rabbits, pigs, sheep) provide more reliable evaluation [220]. Recent studies highlight the osteogenic potential of hybrid scaffolds: Dou et al. [119] showed that 3D-printed gelatin grids with freeze-drying (PHG) promoted significantly greater bone formation than pure printing (PH) or controls (Ctrl) in rat femoral defects (Figure 11A). Similarly, gas-foamed 3D-printed scaffolds (pmPCL44) achieved osteogenesis comparable to CaP-coated scaffolds, with bone detected in both macro- and micropores (Figure 11B) [122]. In large-animal models, Teotia et al. [221] demonstrated that cryogel-infiltrated poly(trimethylene carbonate) (PTMC)/HA (P-HA-CG) scaffolds enhanced bone ingrowth in rabbit calvarial defects over 120 days (Figure 11C). Moreover, vanco-mycin-loaded collagen/hydroxyapatite (COLHA) electrospun layers on SLM titanium implants improved anti-infection efficacy in rats and doubled bone ingrowth in pig femurs (COLHA 47%) versus controls (27%) (Figure 11D) [222].

### 8.2. Challenges and Opportunities

Although the in vivo osteogenic efficacy and long-term safety of multiscale scaffolds have been demonstrated, major barriers hinder their clinical adoption. Regulatory pathways for 3D-printed BTE scaffolds remain complex, as constructs often span multiple categories (medical devices, ATMPs, combination products), creating uncertainty in approval processes [223,224,225]. Hybrid fabrication strategies further exacerbate technical complexity and reproducibility issues, making GMP-standardization and quality control difficult. Calls have been made to shift from conventional “product-based” to “process-based” regulatory frameworks, which may better address manufacturing variability [224]. While ISO and ASTM have initiated standards (e.g., extrusion-based bioprinting, WK65680, WK72274) [226], current guidelines remain nascent and do not yet cover emerging methods such as FRESH, COBICS, TPP, or μMIP-SL. Economic considerations also represent a critical obstacle. The inherent customization and low scalability of 3D-printed scaffolds drive up costs [214]. Moreover, recent MDR reforms in Europe increased documentation and clinical evaluation demands, potentially reducing innovation capacity and accessibility [227]. Without strong health economic evidence and reimbursement strategies, even advanced devices may face limited translation [228].

In summary, the development of next-generation multiscale BTE scaffolds must be seen as a systems-level challenge involving preclinical validation, manufacturing standardization, regulatory coordination, and economic feasibility. Their successful transition from “bench to bedside” will depend on multidisciplinary collaboration and cross-sector innovation.

## 9. Summary and Outlook

### 9.1. Navigating the Mechanics–Biology Trade-Off in BTE Scaffold Design

Multiscale bone tissue engineering scaffold candidates are expected to meet multiple performance criteria simultaneously, including printability, mechanical integrity, biocompatibility, and bioactivity—posing inevitable trade-offs. On one hand, strategies to enhance mechanical strength (e.g., high ceramic loading, dense structural design, and post-printing sintering) often increase material brittleness or result in insufficient surface area for cell adhesion [229]. On the other hand, the pursuit of ultrahigh porosity or highly flexible network structures may improve nutrient exchange and cell infiltration, but significantly compromises the initial mechanical strength and long-term load-bearing capacity of the scaffold [230,231]. To address these challenges, composite, hybrid, and surface-functionalization strategies have become mainstream approaches, aiming to combine mechanically robust components with bioactive phases. For example, titanium mesh/ceramic systems (including allogeneic bone grafts, ABG) [231,232], porous titanium/hydrogel composites [233], and polymer-based/inorganic filler composites (e.g., PCL/TCP [234], PEEK/HA [230]) have demonstrated promising translational potential in preclinical and partial clinical studies.

Notably, beyond the commonly used HA and TCP, novel bioceramics such as monetite [232] and baghdadite [229] are also being explored due to their unique solubility and ion release characteristics, showing potential to enhance osteointegration and bone regeneration. Meanwhile, the emergence of structured meta-biomaterials [235]—engineered through coordinated optimization at both material and geometrical levels—offers a pathway to achieve both mechanical stiffness and cellular friendliness, further advancing the development of functional biomimetic scaffolds [236,237]. In addition, from a biomechanical design perspective, the incorporation of degradable fixation elements or integrated screw or plate systems [238,239] has been proposed to ensure early-stage mechanical stability while allowing gradual replacement by newly formed bone, thereby balancing initial load-bearing needs with long-term tissue regeneration. Overall, future research must seek a more precise balance in the “mechanical-biological trade-off.” A key scientific challenge remains: how to achieve next-generation scaffolds with both high mechanical performance and excellent bioactivity through the multidimensional integration of material composites, structural optimization, surface engineering, and clinical fixation strategies.

### 9.2. In Situ Integration of Macro–Meso and Macro–Micro Structures

Driven by increasing demands for structural precision and functional integration in tissue engineering, in situ fabrication has emerged as a core strategy for the next generation of scaffold manufacturing, The following table summarizes the introduction, strengths, and weaknesses of all the in situ fabrication techniques mentioned above (Table 4). Techniques such as in situ freeze-drying and in situ foaming have disrupted the conventional “fabrication–processing–assembly” paradigm by integrating functional filaments or bioinks to simultaneously achieve structure formation and functional coordination. Future research should prioritize the digital control of intelligent foaming systems, particularly regarding bubble dynamics, gelation kinetics, and environmental responsiveness, to enable precise control over pore architectures and adaptability to physiological conditions.

Simultaneously, in situ mineralization—a critical process for enhancing osteogenic performance—is advancing towards greater biomimicry, controllability, and system integration. Leveraging high-resolution imaging, mass spectrometry, and proteomics, researchers can now gain deeper insights into the microscopic mechanisms of matrix-directed mineralization. The integration of advanced 4D printing with microfluidics-guided hydrogel systems hold promise for achieving spatiotemporal control and functional integration of the mineralization process [240]. Notably, the polymer-induced liquid precursor (PILP) strategy has demonstrated unique advantages in enabling nanoscale infiltration and deep mineral deposition [241]. However, its application at the macroscopic scale still faces challenges in terms of structural uniformity and shape fidelity [242]. Future directions should aim to develop highly integrated, biocompatible, and programmable in situ mineralization platforms, and further integrate them with growth factor carriers and drug delivery systems, achieving intelligent, controllable, and traceable multiscale scaffold fabrication for next-generation personalized bone regeneration.

### 9.3. Construction of Macro–Meso–Micro Multiscale Integrated Technologies

Although significant progress has been made in the fabrication of multiscale-integrated scaffolds, the seamless integration of macro-, meso-, and microscale structures within a single platform remains a substantial challenge. For instance, Huai et al. [243] developed a hierarchically structured scaffold combining a 3D-printed macroscopic framework, mesopores derived from emulsion templating, and microporosity and surface roughness induced by freeze-drying and nanoparticle modification. However, this strategy is currently limited by scalability issues. Future efforts should focus on co-printing of multiple materials and gradient fabrication strategies to enhance interfacial compatibility and functional synergy across scales. In addition, real-time coupling of environmental parameters—such as temperature, humidity, and chemical gradients—during the printing process will be essential for achieving the integration of structure, function, and manufacturing microenvironment, thereby enabling adaptive fabrication. For instance, bio-ink materials used in extrusion-based 3D printing typically contain a high water content and are extremely sensitive to ambient humidity. Under high-humidity conditions, hydrogels tend to undergo excessive water uptake and swelling, resulting in blurred interfaces between filaments and compromised shape fidelity [244]. Conversely, under low-humidity conditions, hydrogels rapidly lose water, leading to volumetric shrinkage, pore collapse, and structural cracking; more critically, cells exposed to dry air are prone to dehydration and death, markedly reducing viability [245]. These challenges have prompted researchers to propose real-time environmental control during the printing process. Bioprinter atmospheric enclosure systems have been developed by Matamoros et al. [246], in which proportional integral derivative (PID) controllers precisely regulate temperature and humidity to ensure hydrogel stability and the maintenance of a consistent cellular microenvironment, thereby improving both structural fidelity and biological performance. Taken together, the convergence of environmental coupling, in situ integration, multiscale coordinated control, and high-throughput fabrication strategies is expected to overcome the current bottleneck of sequential processing, enabling truly integrated biomimetic scaffolds with high performance and scalability for personalized bone regeneration.

## Figures and Tables

**Table 1:** Overview of representative strategies at the mesoscale for scaffold fabrication.

Combining Strategy	Characterization	Mechanical Property	Cell Behavior	Reference
FDM + freeze-drying: A coating was prepared on the surface of FDM-printed PCL scaffolds using a chitosan solution containing wollastonite-hydroxyapatite, applied through a freeze-drying technique.	Macropore size: approximately 500 μm Micropore size: NA	Compressive strength: 3.5–4.9 MPa	NA	[117]
DIW + freeze-drying: Preparation of HA suspension followed by 3D printing via injection extrusion onto a temperature-controlled platform, then freeze-drying and sintering.	Macropore size: submillimeter to millimeter level Micropore size: below 10 µm	Ultimate strength: 22 MPa	Cell adhesion and biocompatibility observed, no quantitative data reported	[118]
3D bioprinting + freeze-drying: PLGA/ HA bioink is extruded through a low-temperature nozzle to fabricate scaffolds, followed by gelatin infiltration and freeze-drying.	Macropore size: 1100 μm Micropore size: 20–500 μm	Compressive strength: 13.7–16.8 MPa	Cell adhesion and in vitro mineralization on PLGA/HA/Gelatin scaffolds were superior to the control group, no quantitative data reported	[119]
DIW+ freeze-drying: Scaffolds were 3D printed with a modified nozzle, infused with bioceramic slurry, bidirectionally frozen, freeze-dried, and sintered into hot dog-like structures	Macropore size: approximately 1 mm Micropore size:18–34 μm	NA	MTT: hot dog-like scaffolds > scaffolds without hot dog structures. Osteogenic genes: Runx2/OCN/OPN/ALP ↑	[120]
3D printing + salt-leaching: CuSO_4_-PLGA ink is rapidly printed via 3D-painting, followed by washing to dissociate and solubilize CuSO_4_ salts and their constituent ions	Macropore size: approximately 200 μm Micropore size: 1–10 µm	Tensile modulus (hydrated): 112.6 MPa → 2.7 MPa (as CuSO_4_ increases 25%→70%)	Greater double-stranded DNA and uniform cell coverage observed in 50% and 70% CuSO_4_-PLGA	[121]
FDM + salt-leaching: The scaffolds were additively manufactured from medical grade polycaprolactone (mPCL) doped with porogen microparticles having an average size of 22 μm, which were subsequently leached to create microscale porosity.	Macropore size: 700 µm Micropore size: 20–70 µm	Young’s modulus: 357.5 ± 31.6 MPa (nonporous mPCL, nmPCL) → 261.6 ± 23.8 MPa (Dual scale porous mPCL with 44% porogen, pmPCL44)	Protein adsorption: pmPCL44 adsorbed 1.8 ± 0.1 μg BSA/scaffold (vs. 0.5 ± 0.3 μg for nmPCL)	[122]
3D bioprinting + sol–gel: 3D-printed Methylcellulose-based hydrogels loaded with bacterial cellulose (BC)-nanofibers (NF)/ superparamagnetic iron oxide nanoparticles(SPIONs) were prepared, ethanol-gelled, then dried via supercritical CO_2_ or freeze-drying to obtain aerogels.	Macropore size:100–600 μm Mesopore size: 10–20 μm	NA	Resazurin test: cell viability > 90% after 24–72 h	[123]
3D printing + TIPS: Embedding porous 3D-plotted polyethylene glycol (PEG) inside PLGA/nHA/1,4-dioxane or PLGA/1,4-dioxane solutions, followed by PEG extraction using deionized (DI) water.	Macropore size: 300 μm, 380 μm, and 460 μm Micropore size: 20–40 μm.	Compressive modulus: 0.37–5.16 MPa ^†^	NA	[124]
FDM + freeze-drying + TIPS: 3D-printed poly (L-lactide) (PLLA) scaffolds were modified via chitosan (CS) coating, freeze-drying, polydopamine (PDA) grafting, and quercetin (Qu) loading to obtain multifunctional hierarchical PLLA/CS-D/Qu scaffolds.	Macropore size: 380–390 μm Nanofiber diameter: 80–600 nm	Compressive strength: 13.1 MPa (PLLA dry) → 15.1 MPa (PLLA/CS-D/Qu dry) Compressive modulus: 0.112 GPa (PLLA dry) → 0.139 GPa (PLLA/CS-D/Qu dry)	Osteogenesis: PLLA/CS-D/Qu’s ALP activity and calcium deposition ~2 × PLLA Osteogenic genes: PLLA/CS-D/Qu’s Runx2, ALP, COL-I, OCN ~4–5 × PLLA	[125]
FDM + gas foaming: PLA resin was blended with a chemical foaming agent (CFA) for filament extrusion, followed by 3D printing.	Macropore size: 200~300 μm Micropore size: 10–60 μm.	NA	Proliferation (NIH3T3): foamed PLA showed ~5 × higher proliferation vs. neat PLA after 10 d	[126]
FDM + gas foaming: PLA and PVA were blended and extruded into filaments, FDM printed, and then gas foamed in a CO_2_-supersaturated hot water bath.	Macropore size: 300–700 μm Micropore size: 0.5–2 μm	Compressive modulus: 86.2 ± 13.4 MPa (unfoamed) → 17.9 ± 5.2 MPa (foamed + etched) Compressive strength: 27.5 ± 5.4 MPa (unfoamed) → 7.6 ± 2.3 MPa (foamed + etched)	NA	[127]

Note: NA = Not available. ^†^: High porosity and nonuniform geometries led to a lack of statistical significance and reduced reproducibility.

**Table 2:** Overview of representative strategies at the microscale for scaffold fabrication.

Combining Strategy	Characterization	Mechanical Property	Cell Behavior	Reference
Extrusion-based 3D printing + electrospinning: The PCL/nHA/ multi-walled carbon nanotubes (MWCNTs) composite ink was first extruded via 3D printing, followed by the fabrication of a ~0.1 mm thick electrospun membrane on the scaffold surface using electrospinning.	Macropore size: 500 μm Nanofiber diameter: 1 μm.	Compressive modulus: 4.40 ± 0.09 MPa (PCL) → 8.23 ± 0.10 MPa (PCL/nHA) → 10.68 ± 0.24 MPa (PCL/nHA/MWCNTs)	Proliferation: CCK-8 ↑ to 464% at day 7 (PCL/nHA/MWCNTs > PCL/nHA > PCL) Osteogenesis: PCL/nHA/MWCNTs ‘s ALP activity ~3 × PCL	[191]
DIW + electrospinning: control PCL (CP) grids were 3D printed and combined with electrospun PCL (PPF) or layered double hydroxide (LDH)/PCL (PLF) nanofiber mats. The nanofiber mats were fixed between printed layers using PCL glue (15%wt in DCM/DMF).	Macropore size: 386 to 459 µm, Nanofiber diameter: 150 to 500 nm.	Young’s modulus (dry): 0.07 ± 0.01 MPa (CP grid) → 0.1 ± 0.06 MPa (PPF) → 0.13 ± 0.05 MPa (PLF) Young’s modulus (wet): 0.06 ± 0.03 MPa (CP) → 0.12 ± 0.05 MPa (PPF) → 0.14 ± 0.07 MPa (PLF)	Viability: MG-63 > 98% after 7 and 14 days (MTT). Mineralization: PLF ‘s Alizarin Red–Ca deposition area ~3 × PPF	[192]
FDM + electrospinning: The hybrid PCL scaffold was constructed by layer-by-layer stacking of 3D-printed PCL filaments and electrospun PCL solution, using a bioprinter equipped with both 3D printing and electrospinning patterns.	Macropore size: 300 µm Nanofiber diameter: 20.2 ± 6.0 µm.	Compression modulus: elastic up to 30% strain; shape recovery after 10 cycles (80% strain)	L-929 fibroblasts: >70% viability in direct and indirect ISO 10993–5 cytotoxicity assays (non-cytotoxic)	[200]
Melt electrowriting (MEW): A high voltage was applied to the nozzle to induce the formation of a Taylor cone from PCL using a custom MEW machine.	macropore size: 100, 200, and 300 μm nanofiber diameter: 4.01 ± 0.06 μm.	Yield force: 100 µm scaffold showed 1.9× (vs. 200 µm) and 2.8× (vs. 300 µm) higher yield force	Seeding efficiency: 55.7% (100 µm) > 24.9% (200 µm) > 19.1% (300 µm) Mineralization: 100 µm scaffold showed 11.6× Ca vs. 300 µm, 2.2× vs. 200 µm at day 21	[201]
3D bioprinting+ alternate soaking: Blue shark collagen was in situ mineralized to form hydroxyapatite; after optimizing conditions, it was mixed with alginate at various ratios to prepare stable bioinks for 3D printing cell-laden hydrogels.	Nanometer-scale apatite crystals were observed on the collagen surface.	NA	Cells exhibit enhanced proliferative capacity in mineralized collagen hydrogel. Qualitative results only; no quantitative percentages reported.	[193]
3D bioprinting+ alternate soaking: Using an acetic acid solution containing type I collagen as bio-ink, a traditional collagen (TC) scaffold was 3D-printed and subsequently mineralized in vitro via the alternate soaking method to obtain a mineralized collagen fiber (MCF) scaffold.	MCF scaffolds covered with nanometer-scale lamellar apatite crystals (Ca/P ratio 1.60–1.72 ≈ bone 1.67).	0° oriented MCF: tensile strength ↑7×, tensile modulus ↑9× vs. TC 45° MCF: tensile strength ↑4×, modulus ↑2× 90° MCF: tensile strength ↑2×, modulus ~same as TC	MCF exhibited superior cell proliferation and in vitro osteogenic induction compared with TC. Qualitative results only; no quantitative percentages reported.	[194]

Note: NA = Not available.

**Table 3:** Commercialized and Approved 3D-Printed Bone Intervention Materials.

Company/Product	Material/AM Process	Indication	Regulatory Status
**Bone Graft Substitutes and Resorbable Scaffolds**			
Dimension Inx–CMFlex^®^	3D extruded CaP composite	Bone defect filling/shaping	FDA 510(k), 2023
Osteopore–Osteoplug^®^/Osteomesh^®^	3D-printed PCL resorbable scaffold	CMF defects, burr hole repair	FDA 510(k), CE
**Patient-Specific CMF Implants**			
Oxford Performance Materials–OsteoFab^®^ PSC/PSF	Laser sintered PEKK	Patient-specific skull/jaw	FDA 510(k)
3D Systems–VSP^®^ PEEK/Metal CMF	PEEK and metal printing	Custom CMF reconstruction	FDA 510(k), 2024
KLS Martin–IPS^®^	L-PBF titanium	Custom cranio-maxillofacial	FDA 510(k), CE
**Spinal/Foot and Ankle Implants**			
Stryker–TRITANIUM^®^	L-PBF porous titanium	Cervical/lumbar fusion	FDA 510(k)
K2M–CASCADIA™ L-3D	Layered porous titanium	Spinal fusion	FDA 510(k)
4WEB Medical–Truss System	L-PBF titanium truss	Spine, foot/ankle, trauma	Multiple FDA 510(k)
DePuy Synthes/LimaCorporate	Trabecular titanium	Spinal cages, hip revision	FDA, CE
**Joint Reconstruction/Revision**			
Stryker–Tritanium^®^	3D-printed porous titanium	Hip/knee arthroplasty	FDA 510(k)
LimaCorporate–Trabecular Titanium^®^	3D-printed titanium	Hip/shoulder revision	CE, FDA shoulder component

Note: Table adapted from publicly available regulatory and industry resources on 3D-printed orthopedic implants. Sources include the FDA 510(k) database, CE-marked device registries, and manufacturer websites (e.g., Stryker, LimaCorporate, 3D Systems).

**Table 4:** Systematic Comparison of Integrated 3D Printing Techniques: Strengths and Weaknesses.

Methods	Concepts and Mechanisms	Strengths	Weaknesses	References
**3D Printing + Freeze-Drying**	Integration with freeze-drying during printing; ice crystal sublimation generates hierarchical porous structures (layered channels, directional pores).	Enables hierarchical porosity (macro + micro), preserves bioactivity, suitable for protein/drug sustained release; low-temperature conditions favor cell/factor preservation.	Complex process, limited layer thickness; low-temperature extrusion inks are difficult to stabilize; limited mechanical strength.	[118,119,120,136,137,138]
**3D Printing + Particulate Leaching/Solvent Casting**	Incorporation of soluble salt particles into printable inks or matrix, followed by leaching to form micropores.	Controllable pore size, simple process, low cost; enables multi-scale porous structures.	Residual solvents may cause cytotoxicity; multiple washing steps required, time-consuming; limited structural stability.	[121,139,146]
**3D Printing + Sol–Gel Self-Assembly**	Sol–gel precursor transformation and self-assembly form nano/micro networks; combined with 3D printing to enhance macromechanical strength.	High porosity, large surface area, potential for functionalization (magnetic response, self-healing, mineralization induction); low-temperature fabrication preserves bioactivity.	Pure sol–gel products have poor mechanical strength; drying/supercritical processing is complex; risk of condensation shrinkage.	[123,140,154,159]
**3D Printing + TIPS**	Cooling or solvent phase separation generates hierarchical pores; extrusion printing allows gradient/core–shell structures.	Controllable pore size/interconnectivity; adaptable for soft/hard tissue; compatible with low-temperature printing to form functional composites.	Complex process, low-temperature scalability issues; risk of residual organic solvents; mechanical performance requires optimization.	[124,143,144,161,162]
**3D Printing + Gas Foaming**	Chemical/physical foaming agents or supercritical gases generate bubble pores; can be embedded in filaments for in situ foaming.	Solvent-free, high porosity; integration with FDM enables macro–micro pore complementarity; suitable for scale-up.	Poor bubble uniformity, tendency to form dense outer layers; insufficient interconnectivity; complex parameter control.	[126,127,141,145,164,165,166,167]
**3D Printing + Electrospinning**	Integration with or post-printing electrospinning; deposition of micro/nanofibers for ECM-like surfaces.	ECM-mimetic topology, enhanced cell adhesion/proliferation; enables multilayer composites.	Weak interfacial bonding, difficult to control fiber orientation; insufficient mechanical strength, limited load-bearing capacity.	[191,192,204]
**3D Printing + In Vitro Mineralization (SBF/PILP)**	Printing collagen or other templates followed by mineralization in simulated body fluid (SBF) or precursor solutions to deposit hydroxyapatite.	High biomimicry, enhances osteogenesis and angiogenesis; allows synergistic fiber alignment + mineralization.	Long process, poor uniformity; difficult to achieve precise spatial control; limited scalability.	[193,194,210,211,212,213]

## Data Availability

The original contributions presented in the study are included in the article, further inquiries can be directed to the corresponding author.

## Abbreviations

The following abbreviations are used in this manuscript:

AM	Additive manufacturing
BTE	Bone tissue engineering
BMP	Bone morphogenetic protein
rhBMP-2	Recombinant human bone morphogenetic protein-2
HA	Hydroxyapatite
TPMS	Triply periodic minimal surface
GBR	Guided bone regeneration
VEGF	Vascular endothelial growth factor
NGF	Nerve growth factor
BDNF	Brain-derived neurotrophic factor
PLA	Polylactic acid
XGMA	Xanthan-glycidyl methacrylate
GelMA	Gelatin methacryloyl
FDM	Fused deposition modeling
SLA	Stereolithography
DLP	Digital light processing
PDA	Polydopamine
CAD	Computer-aided design
CT	Computed tomography
MRI	Magnetic resonance imaging
SLM	Selective laser melting
TPU	Thermoplastic polyurethane
DIW	Direct ink writing
HIPE	High internal phase emulsions
rBMSC	Rat bone marrow mesenchymal stem cell
hBMSC	Human bone marrow mesenchymal stem cell
PLGA	Poly(lactic-co-glycolic acid)
PPF	Poly(propylene fumarate)
TIPS	Thermally induced phase separation
PEG	Polyethylene glycol
LTDM	Low-temperature deposition manufacturing
TCP	Tricalcium phosphate
FFF	Fused filament fabrication
FRESH	Freeform Reversible Embedding of Suspended Hydrogels
AJP	Aerosol Jet Printing
COBICS	Cellular Suspension Ceramic Omnidirectional Bioprinting in Cell Spheroids
TPP	Two-Photon Polymerization
μMIP-SL	Micro Mask-Image Projection Stereolithography
MAO	Micro-arc oxidation
MEW	Melt electrowriting
PTMC	Poly(trimethylene carbonate)
HDPE	High density polyethylene
Qu	Quercetin
CS	Chitosan
ABG	Allogeneic bone graft
